# Prisoners as Users of Digital Health Care and Social Welfare Services: A Finnish Attitude Survey

**DOI:** 10.3390/ijerph18115528

**Published:** 2021-05-21

**Authors:** Teemu Rantanen, Eeva Järveläinen, Teppo Leppälahti

**Affiliations:** 1 Tikkurila Campus, Laurea University of Applied Sciences, Ratatie 22, 01300 Vantaa, Finland; eeva.jarvelainen@laurea.fi; 2 Hyvinkää Campus, Laurea University of Applied Sciences, Uudenmaankatu 22, 05800 Hyvinkää, Finland; teppo.leppalahti@laurea.fi

**Keywords:** digital inclusion, digital exclusion, digital services, prisoners, attitudes, theory of planned behaviour

## Abstract

Prisoners are a group of people with many health and social problems. However, in prisons the use of the Internet is controlled. Thus, prisoners’ access to digital health care and social welfare services is limited. In addition, there are many cognitive and attitudinal barriers to the use of digital health care and social welfare services for prisoners. Cross-sectional survey data (*N* = 225) were collected from eleven prisons in different parts of Finland and analysed using linear regression analysis. The results are consistent with Ajzen’s theory and previous studies on the acceptance of information systems in health care. Prisoners’ behavioural intentions related to the use of digital health care and social welfare services are influenced by their perceptions of their capacity to use digital services, the expectations of their close people and their attitudes, as well as by trust in the Internet and services. In contrast, the age of prisoners’ indirectly affects their willingness to use digital services. The study recommends that prisoners are supported in the use of digital health care and social welfare services by prison staff and other people. Digital skills training is also needed in order to support digital inclusion, especially for older and long-term prisoners.

## 1. Introduction

### 1.1. The Digitisation of Prisons

Prisoners are a group of people with many health and social problems. Prisoners are in poorer health than the rest of the population and most of them have substance abuse problems, and problems related to, for example, released prisoners’ financial situation and housing are common [1]. Furthermore, prisoners’ access to health care and social welfare services is limited. In prisons, the use of the Internet requires permission and it is controlled, and thus prisoners’ access to digital services is also limited. In addition, there are many cognitive and attitudinal barriers to the use of digital health care and social welfare services for prisoners.

The digitalisation of prisons can be justified by the principle of normality and the realisation of human rights [2,3]. In principle, prisoners can be considered to have the same right to digital health care and social welfare services as other citizens. The use of digital services contributes to facilitating the release phase as matters such as housing, work and social benefits can be handled from prison before release [4,5,6]. Digitisation also offers many opportunities for education [7] or for contacting relatives [8]. In addition, McDougall et al. [9] stated that prison technology can promote a prisoner’s sense of worth and personal control when the use of modern technology can transform prisoners’ lives from a state of dependency to self-responsibility. Furthermore, access to digital services supports digital and social inclusion [10,11,12] and thus also contributes to the prevention of the recidivism of released prisoners [9,13,14]. Often, inaccessibility to digital services can exclude a person from society, also causing a digital divide [12,15], and make them second-class citizens in a digitalised society [16]. 

The development of the digitalisation of prisons has progressed significantly in recent times [16,17], and prisoners’ access to digital services have been facilitated in many countries. Knight [17] highlighted the digital technologies available in prisons in different countries (in the UK and across Europe and the USA), their potential and the associated resistance. Various technological solutions—such as e-mail, video visits and video conferencing—are utilised for communication with relatives or authorities. In-cell terminals or laptops also make it possible to participate in distance learning via secure network connections [16,18], in addition to making it possible to conduct official affairs, participate in rehabilitation, apply for work and keep in touch with relatives through white-listing sites [8,19]. Furthermore, with handheld devices (e.g., prison tablets), in some cases prisoners have the opportunity to use elements that entertain them in the everyday life of the prison, such as e-books, movies, games, music, rehabilitation and self-help guidance (see, e.g., [20]). Furthermore, digital kiosks offer an opportunity to make canteen orders and manage daily affairs in prison [2,17,21]. 

An example of the development of prison technology is the Belgium PrisonCloud digital platform, a smart prison concept which combines a wide range of e-services and study, rehabilitation and communication opportunities in prison, supporting the integration of the prisoner into society after release [22,23,24]. Furthermore, in Finland, the development of digital services for prisons has focused on the Hämeenlinna women’s smart prison, which opened in autumn 2020. The prison’s facilities include technology that supports prisoners’ integration into society and acts as a learning environment for a crime-free life [25,26]. Prisoners have the opportunity to contact various officials, participate in rehabilitation and education, apply for work, as well as keep in touch with relatives via cell terminals [19]. Despite these advances, the digitalisation of prisons has been slow in Finland and in other Western countries and access to digital health care and social welfare services and the Internet in general for prisoners is not self-evident, in particular, in closed prisons.

### 1.2. Barriers to the Use of Digital Health Care and Social Welfare Services for Prisoners

There are various barriers to the use of health care and social welfare services in prisons, and prisoners often do not have direct access to these digital services during their imprisonment. Prisoners’ access to the Internet has traditionally been restricted, above all for security reasons [7,27]. It is feared that the use of technology will help a prisoner to organise criminal activity from the prison. Jewkes and Reisdorf [27] stated that digitalisation is changing prison practices and the relationship between the prison and the outside world, reducing prisons’ isolation from the rest of society. In addition, it highlights perceived threats that this new kind of flow of interactive data is much more difficult to manage. Thus, in the prison context, even the use of digital health care and social welfare services is not seen as completely risk-free. 

An individual’s digital skills and attitude are also key issues from the perspective of digital service use and digital inclusion [10,11]. Monteiro et al. [7] showed that many prisoners have, in principle, poor digital skills and low motivation to use electronic services. Digital illiteracy is often a barrier to the digital inclusion of that population [6]. On the other hand, Hustad et al. [28] also recognised that digital personal traits, motivation and digital skills are influential factors in digital inequalities. Reisdorf and Jewkes [29] stated that prisoners have a massive interest in the use of technology, but also have fears and reservations about it. Younger ‘millennials’ have significantly better skills and a desire to use digital services than older, long-term prisoners [27,30].

The inadequate skills of prisoners underline the importance of support from staff in the use of digital services [30,31,32]. This also highlights the importance of prison staff’s attitudes and skills towards digital services. Mufarreh et al. [20] pointed out that staff in prisons with technology are more likely to believe that technology has a positive effect on people in prison. In addition to support from employees, the importance of family and friends in using digital services has also been emphasised [33,34]. On the other hand, for example, Barreiro-Gen and Novo-Corti’s [12] results showed that social support has no significant effect on prisoners’ ICT skills.

Allowing the use of digital services in a prison environment requires staff to have trust in prisoners [7]. In addition to the identified security risks, the lack of trust in prison technology is due to staff attitudes [27]. Prisoners’ trust in digital transactions is also built through experience. Robberechts [35] found that, through the digital platform [22], prisoners ’experiences of privacy increased, although the transaction involved a different collection of personal information when using electronic services. In general, the confidence of prisoners is, in principle, low in relation to other authorities [36] and general confidence in the Internet is weak. Building trust can therefore be seen as one of the key factors in the adoption of prison technology, which enables transactions in digital health care and social welfare services.

### 1.3. The Theory of Planned Behaviour as a Perspective for the Introduction of Digital Services

Previous studies [37,38,39] have shown that, in particular, the technology acceptance model [40] and the theory of planned behaviour [41] are useful approaches for explaining the adoption of new technology in health care. This research focuses on psychological factors influencing behaviour instead of the usability and usefulness of information systems, and therefore the theory of planned behaviour has been chosen as the starting point for the research.

This study examines these barriers to the use of digital health care and social welfare service from the perspective of cognitive social psychology, particularly from the theory of planned behaviour [41]. This theory has been widely applied in the past to explain the use of the digital technology of health care workers, but not in explaining the use of digital health care and social welfare services among prisoners. However, according to previous studies, the theory offers an effective approach, for example, to understanding prisoners’ intentions to participate in an electronic monitoring scheme [42].

The key concept in the theory is behavioural intention, which means a person’s motivation or willingness to exert effort to perform the target behaviour. In prison, the use of the Internet and digital services is externally controlled, and so, in this case, the intention refers, in particular, to the prisoner’s desire or intention to use digital health care and social welfare services in the future, especially during or after the release phase. This means that a person may prefer digital services rather than face-to-face encounters. On the other hand, previous studies suggest that, in a prison context, digital services cannot replace face-to-face interaction [30].

Taking such a perspective in the study emphasises the future use of services rather than current access and the factors that influence it. Similarly, the cognitive approach emphasises a person’s perception of skills and perceived technological control rather than actual skills. According to the theory of planned behaviour, behavioural intention is influenced by perceived behaviour control, subjective norms and attitudes toward that behaviour [41]. In the context of the acceptance and use of health technology, the concept of perceived behavioural control refers to the perception of the availability of skills, resources and opportunities necessary for using the technology and the concept of subjective norms means the perception of important (or relevant) others’ beliefs about the person’s use of system [38,43]. The concept of attitude, in turn, refers to the valuation of a particular object [44], such as a positive or negative value assigned to the use of digital services.

According to previous studies on the adoption of a digital health care service, the user’s belief that a specific service has no security or privacy threats is also an important factor [45,46]. For example, according to the structural model of Gong et al. [47], trust in providers mediates the effect of subjective norms on people’s adoption of online health care services, such as online health consultation services. On the other hand, trust is a complex concept, and it is a different thing to talk about, for example, trust in a particular service, service provider or the Internet in general. Sometimes a distinction is also made between trust in a service and the risks associated with the service [46]. However, according to a study on the utilisation of e-government services, trust in the Internet and trust in a government are closely interconnected, and thus, the perceptions of trustworthiness can be considered as one dimension in this context [48]. In this study, the concept of trust is used in such a general sense that it combines these different dimensions of trust. Consistent with Carter and Bélanger [48], the concept refers to citizens’ perceptions of the trustworthiness of government and technology.

## 2. Materials and Methods

### 2.1. Design

This is a cross-sectional study conducted by questionnaire. The theoretical framework of the study is based on Ajzen’s [41] theory of planned behaviour, and thus the digital inclusion of prisoners is examined using the concepts of behavioural intention, perceived behavioural control, subjective norms and attitudes. Based on previous studies on the adoption of digital services [46], Ajzen’s theory has been supplemented with the concept of trust. 

Several studies have also shown the central importance of age from the perspective of digital inclusion [49,50]. The significance of age has also been observed in studies of prisoners’ digital skills [27,30]. Thus, this study also examines the effect of prisoners’ age on the intention to use digital health care and social welfare services.

**Figure 1:**
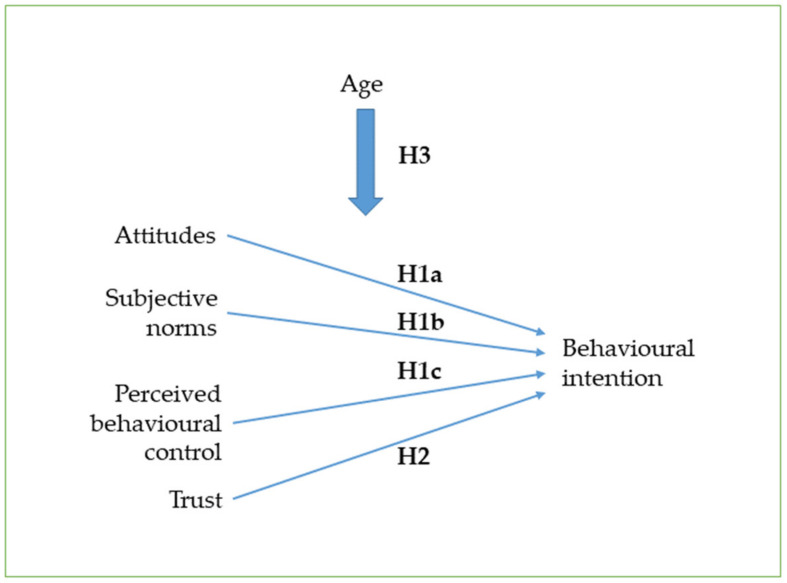
The conceptual model of the study.

In this study, the prisoners’ intention to use digital health care and social welfare services was examined as a dependent variable, while the independent variables used were the perceived behavioural control, subjective norms, attitudes and a person’s age. The association of these independent variables with the intention to use digital health services has also been found empirically in several studies [46]. According to Ajzen [41], the perceived behavioural control, subjective norms and attitudes have a direct effect on behavioral intention. Similarly, trust has been found to directly affect the intention to use new health technologies [46,48]. In contrast, the effect of age on the use of digital services is more complex [46], and so, this study analyses both its direct and indirect effects. On this basis, the conceptual model according to Figure 1 was constructed. 

### 2.2. Aim and Hypotheses

This study examines the attitudes of Finnish prisoners towards the use of digital health care and social welfare services and the adoption of them. The following hypotheses were tested:

**(H1)**  *Prisoners´ behavioural intention related to the use of health care and social welfare services depends on (a) attitudes, (b) subjective norms related to the use of digital services and (c) the perceived behavioural control of the digital services.*

**(H2)**  *A lack of trust in Internet and digital services reduces prisoners’ willingness to use digital health care and social welfare services.*

**(H3)**  *Prisoners’ age reduces their willingness to use digital health care and social welfare services.*

### 2.3. Sample

The sample was purposefully formed to be regionally comprehensive. Thirteen prisons from different parts of Finland were selected for the research sample, but in the end, two prisons were excluded from the study. No permission was obtained to conduct the study in these two prisons. The final sample consisted of a total of eleven prisons. There were both closed prisons (six prisons) and open prisons (five prisons). One closed prison also had an open ward. One closed women’s prison and one prison with a women’s ward were included in the selection. The questionnaire was answered by a total of 225 prisoners. The number of prisoners present in the prisons included in the survey sample was 1131, and thus the response rate was 19.9%.

### 2.4. Measures

The questionnaire contained a total of 24 questions and most of these contained several items. This article focuses on 24 items (Appendix A), which were formed as statements and comprised of Likert-type scale items (ranging from 1 = totally disagree to 5 = totally agree). In addition, the study examined questions related to the respondent’s age and control variables (education level, marital status, number of convictions).

Previous studies on the adoption of health technology have constructed a number of measures for the intention to use health technology and other concepts of the theory of planned behaviour [41], but there are no valid measures related to the intention to use digital health care and social welfare services among prisoners. According to Armitage and Conner’s [51] meta-analyses, previous measures have been related not only to actual intention but also to self-prediction and desire. In this study, the measure for behavioural intention contained five items, which relates, at first, to a person’s general willingness to use digital services and to an assessment of the likelihood of the future use of digital services. The measure also included questions about the desire to apply for social benefits and, on the other hand, to deal with health-related matters via the Internet. One question concerned the desirability of a remote meeting compared with a face-to-face meeting (see the Appendix A).

In this study, the questions related to perceived behavioural control (five items) were concerned with the perceived mastery of digital services and the belief in being able to learn how to use digital services. In addition, it was asked whether the respondent was able to apply for social benefits and the use of self-care programmes. Similarly, questions related to the subjective norm (four items) concern the perceived attitudes of close people towards digital health care and social welfare services in general and issues related to social benefits and health issues in particular. 

The digital attitudes measure (three items), on the other hand, is based on the Australian Digital Inclusion Index [50], although one question included in the original measure was removed during form construction and one inverse question was removed during the analysis phase. The measure of trust was based on Carter and Bélanger’s [48] measures of trust in the Internet and of trust in the state government, and it contained a total of seven items.

In addition, the study examines the respondents’ age and, as control variables, the respondents’ marital status, education level and number of convictions. For the question on marital status, there were four possible answers: married, in a common-law marriage, divorced and unmarried. The education level was measured by using the answer options: no basic education, basic education, secondary education and higher education. In the context of regression, marital status and education level were studied as dummy variables (1 = Married or in a common-law marriage; 1 = At least secondary education). Gender was not included in the analysis due to the small proportion of women.

### 2.5. Procedure

Pre-testing of the questionnaire was conducted among experts by experience with a history of crime and substance abuse (*N* = 11). In this context, respondents were asked for their views on the structure of the form, the ease of answering the questions, the comprehensibility of the questions and the clarity of the answer options. After the pre-testing, some small changes were made to the layout of the form, but the questions themselves did not change.

The research data was collected in paper form between November 2020 and January 2021. Before data collection, the practical implementation of the study was agreed with each prison director. In one prison (with two wards), data collection was carried out by a project worker, and in three prisons it was done by a university student working on the project. In the other prisons, data was collected by prison staff. Responses were returned using envelopes so that prison staff did not see the responses.

### 2.6. Analysis

The sum variables were constructed by averaging the scores from the Likert-scale statements. The internal consistency of the variables was analysed using Cronbach’s alpha coefficient, and the normality of the distributions was examined graphically. The actual analyses were performed parametrically by using Pearson product-moment correlation and linear regression analysis. Before carrying out regression analyses, the validity of the conditions was checked. The normality of the residual distributions and the linearity condition were checked graphically, and the multicollinearity between the independent variables was examined by VIF coefficients.

A linear regression analysis was employed to examine the factors that explained behavioural intention. The first regression model includes all of the independent variables which were examined, and in the second model, the non-affected variables (*p* > 0.05) have been removed one by one. Moderation effects (age*perceived behavioural control, age*subjective norms, age*attitudes, age*trust) were preliminary studied by regression analysis, but no significant effects were found. Mediation effects were analysed using the Sobel test. 

## 3. Results

### 3.1. Respondents

In total, 225 prisoners answered the questionnaire. The average age of the respondents was 37.8 years old (the average age of prisoners serving their sentence was 37.2 years old in Finland in 2019) [52]. The proportion of women was 8.9% (*n* = 20), which corresponds well to the proportion of female prisoners in Finland (about 8% in 2019) [52]. Just under half of the respondents (*n* = 108, 48.0%) were serving their sentences in a closed prison and the remainder were serving them in an open prison.

Of the respondents, 33.0% were married or in a common-law marriage and 56.2% had completed secondary education. The numbers of convictions varied from one (33.3%) to ten or more (14.7%). Of the respondents, 15.1% answered that they have used substance abuse services in the last year and 14.2% said that they have used mental health services.

### 3.2. The Reliabilities of the Measures

Five sum variables were constructed, and their reliability was good (α > 0.8) (see Table 1).

### 3.3. Intention to Use Digital Health Care and Social Welfare Services and Factors That Affect It

Overall, the respondents estimated that they are quite ready to use digital health care and social welfare services in the future. Of the respondents, 62.3% totally or partially agreed with the statement ‘I will use digital social and health services whenever possible in the future.’ Of the respondents, 60.8% totally or partially agreed that they are likely to primarily deal with social and health services in electronic form in the future. However, only 30% of the respondents totally or partially agreed that, in the future, she or he will prefer to have a remote appointment rather than a face-to-face appointment with a social worker, doctor or nurse (see Table 2).

Further, most of the respondents want to apply for social benefits via the Internet in the future. On the other hand, only 39.5% are partially or totally of the opinion that they would like to primarily take care of their health-related issues via the Internet in the future. 

The Pearson correlations (see Table 3) show that prisoners’ intention to use digital health care and social welfare services in the future is connected with the person’s digital attitude, sense of control regarding the use of digital services and also the normative expectations. The correlation between trust and use intention is also strong. Age has a negative correlation with perceived behavioural control. This means that prisoners´ low age is associated with confidence in their ability to use digital services. In contrast, age is not related to their willingness to use digital health care and social welfare services in the future.

The actual analyses were performed parametrically by using linear regression analysis (see Table 4). Before carrying out regression analyses, the validity of the conditions was checked. Additionally, according to the regression model, all the factors examined significantly explain the prisoners’ intention to use digital health care and social welfare services in the future. The effect of subjective norms and perceived behavioural control are strongest. According to Model 1, the effect of age, marital status, education level or number of convictions was not significant, and they have been excluded one by one from the regression model (see Model 2).

In the regression analyses, the VIF coefficients ranged from 1.088 to 1.720, and thus excessive multicollinearity in the regression analysis was not observed.

Age did not correlate significantly with behavioural intention, subjective norms, attitudes, or trust; however, age and perceived behavioural control correlated significantly with each other (Table 3). According to the regression analysis (Table 4), the effect of the respondents’ age was not significant, and age also did not moderate the effect of other independent variables. The mediation analysis using the Sobel test revealed that age has an indirect negative effect on the behavioural intention, and perceived behavioural control was the mediating variable (z = −3.65, *p* < 0.001).

## 4. Discussion

### 4.1. Main Findings

Prisoners’ access to digital health care and social welfare services is limited. In prisons, the use of the Internet requires permission, and it is controlled. Furthermore, there are also many cognitive and attitudinal barriers to the use of digital health care and social welfare services for prisoners. According to this study, the prisoner’s willingness to use digital services would seems to be largely determined by cognitive and attitudinal factors.

Thus, the hypothesis is supported that prisoners’ behavioural intention related to the use of social and health care services depends on the attitudes, perceived behavioural control of the digital services and subjective norms related to the use of digital services. The motivation or willingness to use digital health care and social welfare services would appear to depend on a person’s estimation of how easy it is to use or learn to use digital services (i.e., perceived behavioural control) and the perceived evaluations and expectations of close people (i.e., it depends on the subjective norm). Digital attitudes also play a key role. The study also found a link between trust and intention to use digital health care and social welfare services, and the second hypothesis was supported. A lack of trust in Internet and digital services would appear to reduce prisoners’ willingness to use digital social and health services. According to the results, age did not appear to have a direct effect on the prisoners’ intention to use digital services; however, an indirect effect of age on the intention through perceived behavioural control was found.

### 4.2. Reflection on the Results

Social psychological determinants that affect the adoption of digital health care services have not previously been studied among prisoners, but the results of the study are consistent with the theory of planned behaviour [41] and with previous studies about the acceptance of information systems in health care [38]. Previous studies on the adoption of digital services have shown that trust directly affects the intention to use a service [45,46,47,48], and consideration of the effect of trust can be combined with the theory of planned behaviour [46]. The results of this study are consistent with previous results. In particular, the research supports the view that such a supplemented theory of planned behaviour is also applicable to explaining the digital behaviour of vulnerable people.

According to Helsper [10,11], access, skills and attitudes are key barriers to digital exclusion that are associated with the use of ICT. In a study of prison education, Monteiro et al. [7] also highlighted the importance of trust for digital inclusion. The results of this study are consistent with the findings of Helper and Monteiro et al. Thus, based on the results, the importance of education in digital skills can be emphasised. It is also essential to influence prisoners’ attitudes towards the use of digital services. Building positive attitudes and trust also requires that prison staff support the use of digital health care and social welfare services.

Previous research provides a conflicting view of the importance of close people for the digital inclusion of prisoners. Support from relatives and friends has been seen to be relevant to a person’s intention to use digital services [13,33,34]. On the other hand, according to Barreiro-Gen and Novo-Corti’s [12] findings, families’ and friends’ acceptance and social support has no significant effect on prisoners’ ICT skills. Further, friends with a history of crime can also even maintain a criminal social identity [53]. According to this study, close people play an important role in the digital inclusion of the prisoner and a critical attitude of the prisoner’s close people towards digital services can also act as a barrier to the use of digital health care and social welfare services. Thus, in promoting the digital inclusion of prisoners, attention should be paid not only to the support provided by the staff but also to the importance of the people close to them. However, in the context of peer associations, the importance of ex-prisoners whose desistance from crime is sufficiently advanced must be emphasised. 

According to Jewkes and Reisdorf [27], older prisoners, as well as long-term prisoners, are a group whose use of technology is associated with specific challenges, or even resistance, due to their incompetence. On the other hand, millennials are often accustomed to navigating digital society and, due to their young age, are very smooth users of digital services [30]. However, in this survey, age does not appear to be a relevant factor in terms of use intention, although it does have a significant effect on the sense of control. In this sense, it is justified to provide targeted support for older prisoners in regard to the use of digital services.

According to previous studies, prisoners emphasise the importance of face-to-face encounters and support from a prison employee instead of just digital encounters [7,30,32]. When technology is added to the prison context, remote encounters must not replace these face-to-face encounters [30]. The results of this study are consistent with previous studies. In the case of health care services in particular, some respondents prefer face-to-face encounters rather than remote encounters. In contrast, when applying for social benefits, the majority of respondents prefer digital transactions. From an overall perspective, prisoners would appear to be quite willing to introduce digital health care and social welfare services.

### 4.3. Limitations

The study has been carried out in eleven prisons across Finland and the regional coverage of the study can be considered reasonably good. On the other hand, the study does not allow the examination of differences between prisons, although it is obvious that different practices in prisons can have a significant impact on access to the Internet and digital services. In addition, when generalising the results, it must be taken into account that only a small proportion of Finnish prisoners responded to the survey. It is also possible that less of those prisoners who do not use the Internet have responded than other prisoners. On the other hand, the use of a paper questionnaire partially reduces the related distortion of the results. As the survey was conducted using a Finnish-language form, those prisoners who do not speak Finnish or have poor language skills were practically excluded from the survey. It is likely that among these prisoners, digital literacy is also lower and thus the risk of digital exclusion is higher.

The research is limited to Finnish prisons. The identified barriers to the use of digital services are largely consistent with international studies on digital inclusion [7,10,11] and the digitalisation of prisons [24,27]. However, the results of this study cannot be directly generalized outside Finland.

This is a cross-sectional study. Such a research design makes visible the existence of statistical relationships between different variables. However, it does not provide a reliable indication of causal relationships between examined variables. The reliabilities of all the variables used in the study were quite good. Similarly, the explanatory rates of the regression model were very high. In this sense, the explanatory model of the study can be considered quite reliable.

## 5. Conclusions

Prisoners are a group of people with inadequate access to digital health care and social welfare services, and their use of the Internet is controlled. In addition, barriers to the use of digital health care and social welfare services include a lack of digital skills and the associated lack of perceived control, the prisoner’s attitudes and the attitudes of the people close to him or her, as well as a lack of trust in digital services. The study recommends paying attention to digital support. Informal support from a prisoner’s close people is also crucial. Emphasis should be placed on supporting the digital skills of elderly and long-term prisoners in particular. In the critical phase of release, the prisoner’s access to digital health care and social welfare services is emphasised as enabling smooth integration into society. 

Overall, prisoners would seem to be quite willing to make use of digital health care and social welfare services. However, replacing face-to-face encounters with digital transactions also raises opposing views. Ideally, the prisoner should have both access to digital services and the opportunity for social support and face-to-face encounters with health care workers. This requires that prisoners be seen as a group of people whose need and right to diverse health care and social welfare services is recognised.

## Figures and Tables

**Table 1:** Variables included in the research design and their means, SD and reliability.

Variable	Items	*N*	Mean	*SD*	Cronbach *α*
Intention to use digital services	5	221	3.40	1.03	0.866
Perceived behavioural control	5	221	3.69	1.04	0.908
Subjective norms	4	219	3.53	0.92	0.832
Attitudes	3	221	3.98	0.99	0.803
Trust	7	222	3.37	1.12	0.967
Age	1	193	37.8	11.7	-

**Table 2:** The distributions of the responses regarding behavioural intentions.

Statements	*N*	I Totally Disagree (%)	I Partially Disagree (%)	I Neither Agree nor Disagree (%)	I Partially Agree (%)	I Totally Agree (%)
I will use digital social and health services whenever possible in the future.	223	7.2	5.8	24.7	31.4	30.9
I am likely to primarily deal with social and health services in electronic form in the future.	222	8.1	8.1	23.0	28.8	32.0
When I need to talk to a professional in the future, I will prefer to meet remotely rather than face to face, if at all possible.	223	28.7	13.9	27.4	15.7	14.3
I want to manage matters related to my social benefits via the Internet in the future.	222	6.8	9.9	25.2	23.4	34.7
I would like to primarily take care my health-related issues via the Internet in the future.	223	15.7	13.9	30.9	18.8	20.6

**Table 3:** Pearson correlations.

Variable	Intention to Use Digital Services	Perceived Behavioural Control	Subjective Norms	Attitudes	Trust	Age
Intention to use digital services	1					
Perceived behavioural control	0.712 *p* < 0.001 *N* = 219	1				
Subjective norms	0.696 *p* < 0.001 *N* = 219	0.485 *p* < 0.001 *N* = 217	1			
Attitudes	0.493 *p* < 0.001 *N* = 217	0.444 *p* < 0.001 *N* = 217	0.365 *p* < 0.001 *N* = 216	1		
Trust	0.643 *p* < 0.001 *N* = 219	0.539 *p* < 0.001 *N* = 218	0.565 *p* < 0.001 *N* = 217	0.379 *p* < 0.001 *N* = 218	1	
Age	−0.093 *p* = 0.201 *N* = 191	−0.261 *p* < 0.001 *N* = 191	−0.072 *p* = 0.322 *N* = 189	−0.128 *p* = 0.080 *N* = 189	−0.120 *p* = 0.096 *N* = 192	1

**Table 4:** Linear regression analysis (dependent variable: intention to use digital services).

Model 1^1^						
**Independent Variable**	***B***	**SE**	**Beta**	***t***	***p***	***VIF***
(constant)	−0.938	0.346	−	−2.707	0.008	−
Perceived behavioural control	0.326	0.063	0.335	5.154	<0.001	1.599
Subjective norms	0.443	0.068	0.403	6.524	<0.001	1.441
Attitudes	0.166	0.062	0.153	2.656	0.009	1.262
Trust	0.196	0.061	0.217	3.246	0.001	1.690
Age	0.003	0.005	0.033	0.521	0.603	1.489
Marital status	−0.032	0.113	−0.015	−0.281	0.780	1.088
Education level	0.135	0.109	0.069	1.230	0.221	1.201
Number of convictions	0.009	0.012	0.045	0.785	0.434	1.234
Model 2^2^						
**Independent Variable**	***B***	**SE**	**Beta**	***t***	***p***	***VIF***
(constant)	−0.547	0.198	−	−2.763	0.006	−
Perceived behavioural control	0.368	0.048	0.374	7.700	<0.001	1.650
Subjective norms	0.423	0.054	0.375	7.816	<0.001	1.606
Attitudes	0.139	0.047	0.128	2.960	0.003	1.319
Trust	0.161	0.046	0.174	3.515	0.001	1.720

^1^ R^2^ = 0.664, Adjusted R^2^ = 0.643, F = 31.4; *p* < 0.001; N = 135. ^2^ R^2^ = 0.704, Adjusted R^2^ = 0.698, F = 123.1; *p* < 0.001; N = 211.

## Data Availability

The respondents represent a vulnerable group of people. Sharing the data is not in accordance with the consent provided by the participants.

## Appendix A

**The statements included in the measures.**

**Intention to use digital services**

I will use digital social and health services whenever possible in the future.

I am likely to primarily deal with social and health services in electronic form in the future.

When I need to talk to a professional (social worker, doctor, nurse etc.) in the future, I will prefer to meet remotely rather than face to face, if at all possible.

I want to manage matters related to my social benefits via the Internet in the future.

I would like to primarily take care my health-related issues via the Internet in the future.

**Perceived behavioural control**

The use of digital services is easy for me.

The use of various digital social and health services is completely under my control.

I am confident that I can easily learn how to use new digital services.

I am able to apply for various social benefits electronically (e.g. unemployment assistance, labour market assistance, sickness assistance, subsistence assistance, housing assistance).

I am able to use various digital self-care programs.

**Subjective norms**

People who are important to me welcome digital social and health services.

People in my immediate circle of civilians think that I should primarily use digital services.

People close to me (in civilian circles) take care of their social benefits via the Internet.

People close to me (in civilian circles) often take care of their health issues via the Internet.

**Attitudes**

Computers and technology give me more control over my life.

I am interested in being able to access the Internet wherever I am.

I go out of my way to learn everything I can about new technology.

**Trust**

The Internet has enough safeguards to make me feel comfortable using it to interact with social and health care services online.

I feel assured that legal and technological structures adequately protect me from problems on the Internet.

In general, the Internet is now a robust and safe environment in which to transact with social and health care services.

I think I can trust social and health care services.

Social and health care services can be trusted to carry out online transactions faithfully.

In my opinion, social and health care services actors are trustworthy.

I trust social and health care services to keep my best interests in mind.
